# Wnt Pathway Activity in Breast Cancer Sub-Types and Stem-Like Cells

**DOI:** 10.1371/journal.pone.0067811

**Published:** 2013-07-04

**Authors:** Rebecca Lamb, Matthew P. Ablett, Katherine Spence, Göran Landberg, Andrew H. Sims, Robert B. Clarke

**Affiliations:** 1 Breakthrough Breast Cancer Research Unit, University of Manchester, Manchester, England; 2 Breast Biology Group, University of Manchester, Manchester, England; 3 Applied Bioinformatics of Cancer, Edinburgh Cancer Research Centre, Edinburgh, Scotland; Health Canada and University of Ottawa, Canada

## Abstract

**Introduction:**

Wnt signalling has been implicated in stem cell regulation however its role in breast cancer stem cell regulation remains unclear.

**Methods:**

We used a panel of normal and breast cancer cell lines to assess Wnt pathway gene and protein expression, and for the investigation of Wnt signalling within stem cell-enriched populations, mRNA and protein expression was analysed after the selection of anoikis-resistant cells. Finally, cell lines and patient-derived samples were used to investigate Wnt pathway effects on stem cell activity *in vitro*.

**Results:**

Wnt pathway signalling increased in cancer compared to normal breast and in both cell lines and patient samples, expression of Wnt pathway genes correlated with estrogen receptor (ER) expression. Furthermore, specific Wnt pathway genes were predictive for recurrence within subtypes of breast cancer. Canonical Wnt pathway genes were increased in breast cancer stem cell-enriched populations in comparison to normal breast stem cell-enriched populations. Furthermore in cell lines, the ligand Wnt3a increased whilst the inhibitor DKK1 reduced mammosphere formation with the greatest inhibitory effects observed in ER+ve breast cancer cell lines. In patient-derived metastatic breast cancer samples, only ER-ve mammospheres were responsive to the ligand Wnt3a. However, the inhibitor DKK1 efficiently inhibited both ER+ve and ER-ve breast cancer but not normal mammosphere formation, suggesting that the Wnt pathway is aberrantly activated in breast cancer mammospheres.

**Conclusions:**

Collectively, these data highlight differential Wnt signalling in breast cancer subtypes and activity in patient-derived metastatic cancer stem-like cells indicating a potential for Wnt-targeted treatment in breast cancers.

## Introduction

WNT proteins are a family of secreted, glycosylated, and palmitoylated peptides which function in diverse processes such as embryonic induction, generation of cell polarity, and cell fate specification [1]. Aberrant activation of Wnt signaling has been described in a number of human cancers including colorectal cancer, ovarian cancer and breast cancer [2], [3], [4], [5], [6], [7]. β-catenin expression has been associated with poor prognosis in breast cancer patients in a number of studies [8], [9] and is enriched in basal-like breast cancer [6]. Furthermore loss of negative pathway regulators such as the extracellular inhibitor of WNT signaling, secreted Frizzled-related protein 1 (*sFRP1*), is found in many breast tumors and is associated with poor prognosis [3], [10]. Down regulation of the inhibitor Dickkopf 1 (*DKK1*) in a lung metastases derived MCF7-LM cell line demonstrates the importance of Wnt regulation in the metastatic process in breast cancer [11]. Collectively these data suggest that WNT pathway de-regulation within the breast contributes to cancer formation and metastasis.

Recent studies suggest breast cancer initiation and recurrence may be regulated by a small population of cells within the tumor, either stem cells or cells that exhibit stem-like properties [12]. Transplantation experiments using immunocomprimised mice, showed that as few as 100 human breast cancer cells with the cell surface markers CD44^+^CD24^−/low^ were tumorigenic and could be serially passaged to generate new tumours [13].

Cells isolated from human breast cancers marked by CD44^+^CD24^−/low^ lineage are anoikis-resistant and capable of self-renewal as mammosphere (MS) colonies providing a link between MS and cell surface markers that enrich for tumorgenic cells [14], [15].

Expression of Wnt1 in human mammary epithelial cells increases stem cell self renewal, resistance to apoptosis and failure to senesce [16]. More recent work using the MMTV-WNT-1 mouse model has identified an expanded mammary stem cell (SC) pool from a population of committed luminal progenitors indicating that Wnt-1 activation induces the appearance of aberrant progenitor cells, and suggest that both mammary stem and progenitor cells can serve as the cellular targets of WNT-1-induced tumorigenesis [17].

WNT pathway activation increases radio resistance of progenitor cells in the mouse mammary gland and human breast cancer cell lines [18], [19], which implicates the WNT pathway in resistance to current anti-cancer therapies, potentially through the regulation of stem and progenitor cell populations.

In this study we investigated the WNT pathway both in whole cell populations and stem-like cells of breast cell lines and patient-derived metastatic breast cancer samples. WNT pathway gene expression correlates with estrogen receptor (ER) expression and molecular sub-type, and some genes predict prognosis. WNT signalling was found to be activated in breast cancer stem-like cells compared to normal stem-like cells. Finally, we show that WNT pathway inhibition preferentially reduces stem-like cell activity in patient-derived metastatic breast cancer compared to normal cells. Collectively, these data suggest potential of the WNT-targeted therapeutics in breast cancer.

## Methods

### Cell Lines

Six normal breast (HB4A, MRSV4.4, MRSV1.7, MCF10A, MCF10F, 226L-U19), six ER+ve breast cancer (BT474, MCF7, MCF7-HER2-18, MDA-MB361, T47D and ZR75-1) and five ER-ve cancer (BT20, Hs578T, MDA-MB231, MDA-MB468 and SKBR3) cell lines were used in this study.

### Primary Normal Human Breast Cells

Histologically confirmed normal breast tissue was obtained from 3 premenopausal patients undergoing fibroadenoma excision or breast reduction surgery (n = 3; mean age 38). Normal breast tissue was prepared and purified as previously described [20]. Briefly, tissue samples were dissected into 3- to 5-mm cubes and digested for 16–18 hours at 37°C in serum-free Dulbecco’s modified Eagle Medium (DMEM; Gibco) containing 200 U/mL type 3 collagenase (Worthington Biochemical Corporation), 5 mg/ml pronase (Roche) and 10x antibiotic/antimycotic (Invitrogen). The cells were then washed, collected by centrifugation at 1000 *g* and filtered to obtain a single-cell suspension, which was verified microscopically. CD45 positive cells were removed using anti-CD45 magnetic beads (Miltenyi). CD45 negative cells were then collected and resuspended in mammosphere culture medium.

### Patient-derived Metastatic Breast Cancer Cells

Samples were collected from patients (ER+ve n = 3 and ER-ve n = 3) with metastatic breast cancer (Table 1). Tumour samples were prepared and purified as previously described [15]. Briefly, metastatic fluid was collected and centrifuged at 2000 g from 5 minutes and responded in PBS. Blood cells were removed by centrifugation through Lymphoprep solution (Axis Shield), followed by removal of CD45 positive cells using anti-CD45 magnetic beads (Miltenyi). CD45 negative cells were then collected and resuspended in mammosphere culture medium.

**Table 1:** Primary metastatic breast cancer samples.

Sample ID	Histology	Source	Grade	ER	PgR	Her2
BB3RC30	IDC	PE	2	Pos	Pos	UN
BB3RC33	ILC	AS	2	Pos	Pos	Pos
BB3RC36	IDC	PE	2	Pos	Pos	UN
BB3RC37	IDC	PE	UN	Neg	Neg	Neg
BB3RC38	IDC	AS	3	Neg	Neg	Neg
BB3RC39	IDC	PE	3	Neg	Neg	Neg

Details of primary breast cancer samples used in this study. **UN** unknown, **PE** pleural effusion, **AS** Ascites sample **IDC** invasive ductal carcinoma, **ILC** invasive lobular carcinoma, **ER** oestrogen receptor alpha, **PgR** progesterone receptor, **Neg** negative, **Pos** positive.

### Ethics Statement

Any experimental research reported in the manuscript has been performed with the approval of an appropriate ethics committee and in compliance with the Helsinki Declaration. All samples were collected with informed written consent. Normal breast tissue was collected with approval from South Manchester Research Ethics Committee (COREC#05/1403/159). Patient derived metastatic breast cancer cells were collected with approval from Tameside and Glossop Local Research Ethics Committee (COREC # 05/Q1402/25).

### Mammosphere Culture

A single cell suspension was created from cell lines grown in monolayer culture using enzymatic digestion (Trypsin EDTA) followed by manual disaggregation (25 Gauge needle). Patient-derived breast cells did not require the addition of trypsin, a single cell suspension was created using manual disaggregation alone. Single cells from cell lines and patient derived cells were plated at a density of 500 cells/cm^2^ in non-adherent conditions, in culture flasks coated with (2-hydroxyethylmethacrylate) (poly-HEMA [Sigma]). Culture of cells in these conditions allows the survival of stem-like cells and subsequent spheroid growth from a single cell using both cell lines and primary tissue samples. Mammospheres grow at a similar rate when plated as single cells or at higher densities indicating that mammospheres are truly clonal structures and not formed through aggregation [21], [22].

#### Anoikis resistant cells

were collected after 12 hrs (RNA) or 24 hrs (protein) in non-adherent, mammosphere culture. As described above, single cell suspensions were obtained and cultured in non-adherent conditions at a density of 500 cells/cm^2.^ Anoikis resistant cells were then collected by centrifugation at 1800 rpm for 2 minutes. Prior to RNA/protein extraction cells were incubated with 100 ul of Dead cell Removal micro-beads and dead cells removed using an MS column and MACS Seperator (Miltenyi Biotech). ***Mammospheres*** (MS) were counted after culture for 7 days in MS medium (DMEM: F12 medium supplemented with B27 without vitamin A [diluted 1∶ 50; Gibco]) and mammary epithelial growth medium aliquot of gentamicin/amphotericin-B and recombinant human epidermal growth factor (EGF), (SingleQuot) (Lorne Laboratories). Spheres over 50 µM were counted and the percentage of cells plated which formed spheres was calculated and is referred to as the percentage mammosphere formation units (%MFU). All cells were maintained in a humidified incubator at 37°C at an atmospheric pressure in 5% (v/v) carbon dioxide/air.

#### Wnt pathway inhibition

MCF10a, MCF7, MDA-MB-231, primary human normal breast cells and primary human invasive breast cancer cells were plated into MS culture and treated with a single dose of human recombinant DKK1 (R and D systems) at increasing concentrations (0–100ng/ml).

#### Wnt pathway activation

MCF10a, MCF7, MDA-MB-231, Patient-derived normal and metastatic breast cancer cells were plated into MS culture and treated with a single dose of mouse recombinant WNT3A (R and D systems) at increasing concentrations (0–50 ng/ml).

### RNA Extraction and Real-time Quantitative PCR Assays of Breast Cell Lines

Total RNA was extracted from the sixteen cell lines harvested at log phase, using the RNeasy® Plus (Qiagen) kit following the manufacturer’s protocol. For real-time quantitative PCR (qPCR), mRNA was reverse-transcribed into cDNA using an ABI RT kit (Applied Biosystems). qPCR primers and probes were designed using Roche Probe Finder Design Software (Roche Applied Sciences) and reactions performed using the ABI PRISM 7900 Sequence Detection System instrument and software (Applied Biosystems). The reactions were incubated in a 384-well optical plate at 95°C for 10 min, followed by 40 cycles of 95°C for 15 s and 60°C for 10 min. Experiments were performed in triplicate for each sample. Gene expression was normalized to internal control *SDHA* (succinate dehydrogenase), *YWHAZ* (tyrosine 3-monooxygenase/tryptophan 5-monooxygenase activation protein, zeta polypeptide).

### RNA Extraction of Monolayer and AR Breast Cells

Cells were collected from 226L-U19, HB4a, MCF10a, MCF7, T47D and SKBR3 cell lines cultured in monolayer (pre-incubated with MS media for 12 hrs) and from viable AR cells. RNA was extracted using the RNeasy® Plus Mini (Qiagen) following the manufacturers protocol. In addition to the gDNA eliminator spin column provided in the RNeasy kit, an on-column DNase digestion was performed (RNase-Free DNase Set; Qiagen) to ensure maximum removal of DNA. RNA was eluted and the concentration measured using a GeneQuant machine (Amersham Biosciences). RNA integrity was assessed by microanalysis (Agilent Bioanalyser) and amplified using the WT-Ovation™ Pico RNA Amplification System (NuGEN) following the manufacturers protocol which employs SPI™ amplification (linear isothermal DNA amplification process) to amplify the RNA about 15,000-fold.

### Custom Gene Expression Microarray Analysis of Monolayer and AR Breast Cells

Custom microarray chips were designed using Agilent technology. cDNA was fluorescently labelled using the FL-Ovation™ cDNA Fluorescent Module kit (NuGEN) with a single tag (Cy™3) which incorporates into the cDNA. The fluorescently tagged cDNA was loaded onto a microarray slides and the Agilent microarray chips attached. Slides were incubated at 65°C for 40 hours in a hybridisation oven to allow hybridisation to occur. The chips were then washed and scanned using an Agilent scanner.

### Primary Breast Cancer Microarray

Affymetrix gene expression data representing a total of 1107 primary breast tumors from six previously published microarray studies [23], [24], [25], [26], [27], [28] were integrated as described previously using ComBat [29] to remove batch effects [30]. Centroid prediction [31] was used to assign the tumors from each dataset to the five Norway/Stanford subtypes (Basal, Luminal A, Luminal B, ERBB2 and Normal-like [32].

### Gene Expression Analysis

Centred average linkage clustering of cell lines, monolayer/anoikis resistant cells and integrated tumour datasets was performed using Cluster [33] and heatmaps generated using TreeView programs as described previously [34].

### Accession Numbers

Genbank accession numbers from NCBI, of genes used in microarray are provided below. [Genbank: NM_004655, NM_001904, NM_012242, NM_000125, NM_012193, NM_003507, NM_003508, NM_002093, NM_030915, NM_001130713, NM_002335, NM_002336, NM_003012, NM_003013, NM_003014, NM_005985, NM_001083962, NM_001204869, NM_003881, NM_005430, NM_025216, NM_003394, NM_030761, NM_001256105, NM_030775].

### Protein Analysis

Western blotting was carried out as previously described [35]. Antibodies used in this study were anti unphoshorylated B-catenin (Upstate, Millipore) *AXIN2* (Cell signalling) *LEF1* (Cell signalling) and *DKK1* (abcam) and *B-ACTIN* (abcam).

### Statistical Analyses

Normally distributed data was analysed using analysis of variance (ANOVA) to determine significant differences of DKK1 treatment followed by individual comparisons to control (0 ng/ml) using the independent T-test. Data which deviated from the normal distribution was analysed using Kruskall-wallis followed by individual comparisons to control (0 ng/ml) using the Mann Whitney U test. *Gene expression*: data was analysed using two tailed students’t-tests.

## Results

### Activation of Wnt Signalling in Breast Cancer Cell Lines

**Figure 1:**
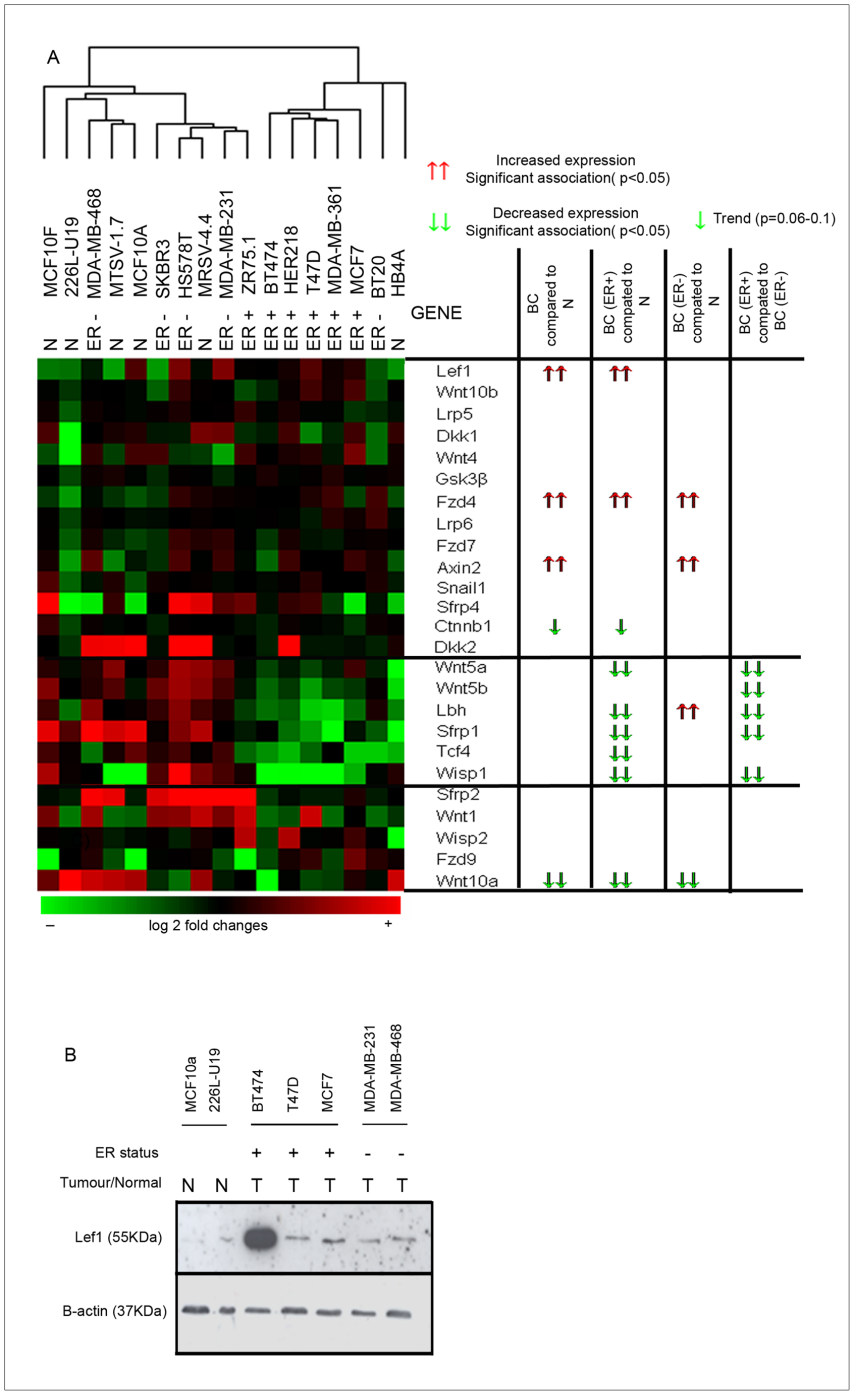
Wnt signalling in normal breast cell lines (N), ER-ve (T−) and ER+ve (T+) breast cancer cell lines. A) mRNA expression was normalised to house keeper genes. Cluster analysis was performed and data displayed in a heatmap: decreased (green) or increased (red) expression compared to the mean mRNA expression. (Red) ↑↑ Indicates significant increased expression (<0.05) (red) ↑ indicates a trend towards increased expression. (Green) Indicates significant increased expression (<0.05) (green) indicated a trend towards increased expression. B) Protein expression of Lef 1 and B-actin (housekeeper) in normal breast cell lines (N), ER-ve (T−) and ER+ve (T+) breast cancer cell lines.

Comprehensive analysis of WNT pathway mRNA expression across a large panel of breast cell lines was performed, which revealed cell type specific gene expression within the cell lines tested (Figure 1A). Well established downstream targets, *AXIN2* and *LEF1* (Figure 1A) showed higher expression in breast cancer cell lines suggesting activation of canonical WNT signalling. Consistent with its gene expression level, LEF1 protein was higher in five breast cancer cell lines compared to two normal cell lines (MCF10A and 226LU19), providing further evidence that WNT signalling is active in breast cancer cell lines (Figure 1B). Interestingly, we observed high levels of LEF1 in the HER2 expressing cell line BT474. HER2 is known to activate β-catenin activity which may account for this increase in the downstream target LEF1 [36]. A WNT receptor and a WNT ligand were also dysregulated in breast cancer cell lines, *FZD4* showed higher expression in breast cancer cell lines whilst *WNT10A* expression was decreased. The expression pattern of WNT pathway genes largely clustered the cell lines by estrogen receptor-α (ER) status; ER+ve cell lines predominately expressed the downstream target *LEF1* whilst ER-ve cell lines express another downstream gene *AXIN2* (Figure 1A). As reported by others, *WNT5A, LBH, WISP1* and *TCF4* were expressed at lower levels in ER+ cell lines compared to ER-ve cancer but also compared to immortalised normal breast cell lines (Figure 1A), perhaps reflecting their ER-ve status. Independent of ER status, overall WNT pathway signalling assessed by the canonical downstream target genes *AXIN2* and *LEF1* is higher in breast cancer cell lines compared to normal breast cell lines.

### Prognostic Value of WNT Expression in Breast Cancer

**Figure 2:**
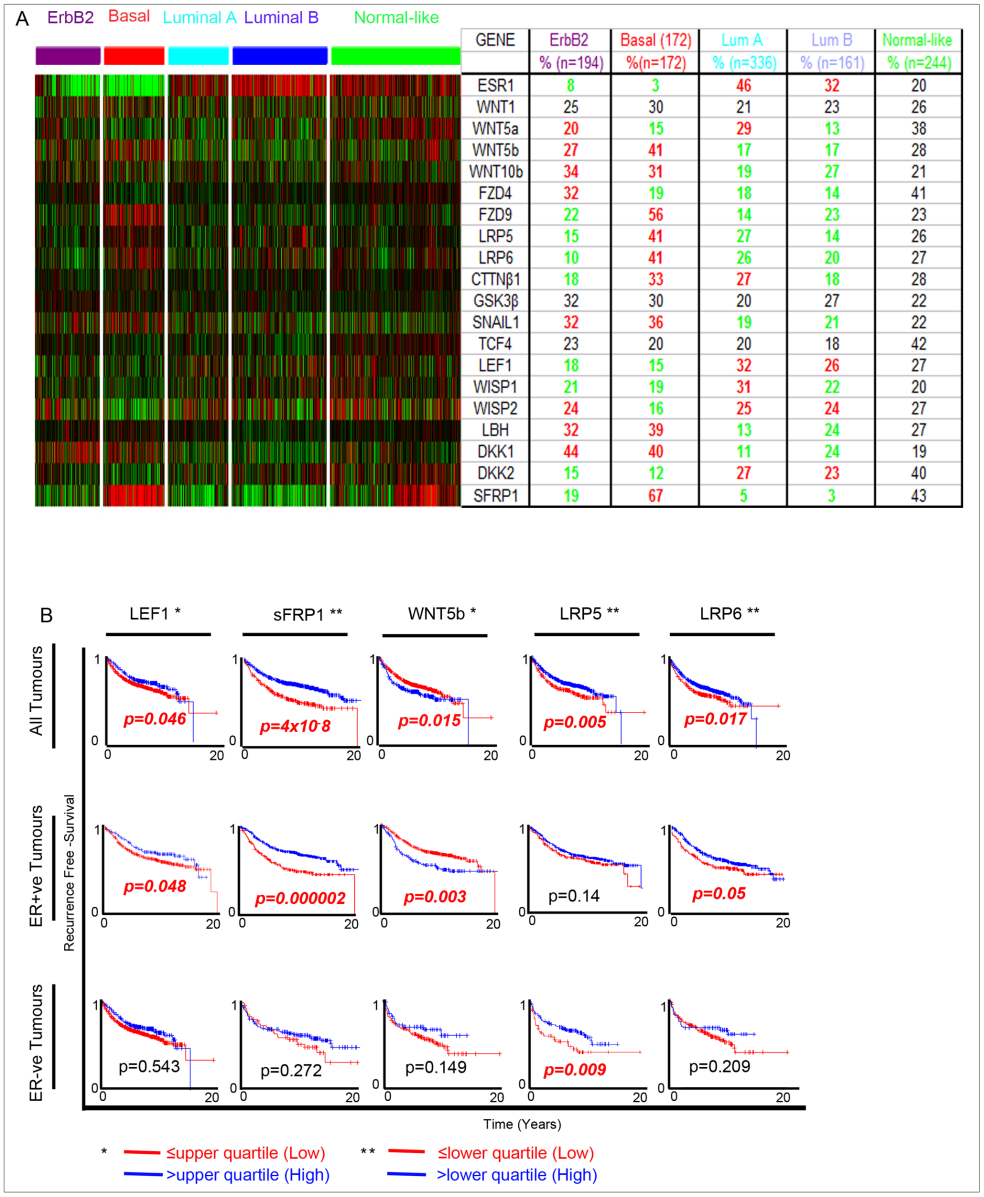
Analysis of mRNA expression in primary human breast cancer samples. Expression data was combined from multiple studies into a combined cohort of ERBB2 (n = 194) Basal (172) Luminal A (n = 336) and Luminal B (161) and normal-like (n = 244) subtypes of breast cancer. A) mRNA expression data was displayed in a heatmap: decreased (green) or increased (red) expression compared to the mean mRNA expression. % of samples with high gene expression are detailed and denoted as green when expression is decreased and red when expression is increased. B) Kaplan Meier plots Wnt gene expression show the years of recurrence free survival, where red indicates lower gene expression and blue indicates higher gene expression. * Red ≤upper quartile: Blue >upper quartile ** Red ≤lower quartile: Blue >lower quartile. P values were generated and significant results were highlighted in red.

To assess the clinical importance of WNT pathway genes, we performed analysis of a large cohort of breast cancer tumours comprising six publically available gene expression data set [30]. Analysis of WNT signalling gene expression within defined subtypes of breast cancer showed variation in gene expression across the subgroups. Confirming the results observed using cell lines (Figure 1) we observed higher expression of *LEF1*, suggesting WNT activation in Luminal, ER+ve breast cancer patients (Figure 2A). A number of genes were differentially expressed between Luminal and Basal/ErbB2 breast cancer, such as *WNT5B, WNT10B* and *DKK1*. A number of genes were highly expressed specifically within the basal subtype of breast cancer, *LRP5, LRP6*, and *sFRP1* (Figure 2A). Analysis of these genes showed significant associations to recurrence free survival within breast cancer patients. The most significant association was with *sFRP1*, where decreased expression predicts early recurrence in ER+ve breast cancer, most likely due to lack of Wnt inhibition. Lower levels of *Lef1* were also predictive of recurrence in breast cancer within the ER+ve subgroup. It might be expected that higher levels of Lef1 would correlate with recurrence, however lower Lef1 expression may identify a subgroup of ER+ve tumours that have a basal-type phenotype, based upon their Wnt gene expression. This subgroup within ER+ve tumours can also be observed with analysis of Wnt5b, where high expression predicts early recurrence, and where high expression is a marker of the basal-type phenotype. LRP5 and LRP6 although demonstrating a very similar expression profile within breast tumours have very different effects on recurrence. Low expression of LRP6 in ER+ve tumours was predictive of early recurrence, whilst low levels of *LRP5* was predictive of early recurrence in ER-ve breast cancer (Figure 2B). LRP5/6 are co-receptors for WNT ligands, previously thought to act similarly to activate downstream signalling. Recent research suggests that they may actually function differently, dependant upon physiological conditions and the type and availability of WNT ligand [37], [38]. Further investigations will be needed to determine their precise roles in breast cancer.

### Wnt Pathway Activation in Breast Cancer Stem-like Cells

**Figure 3:**
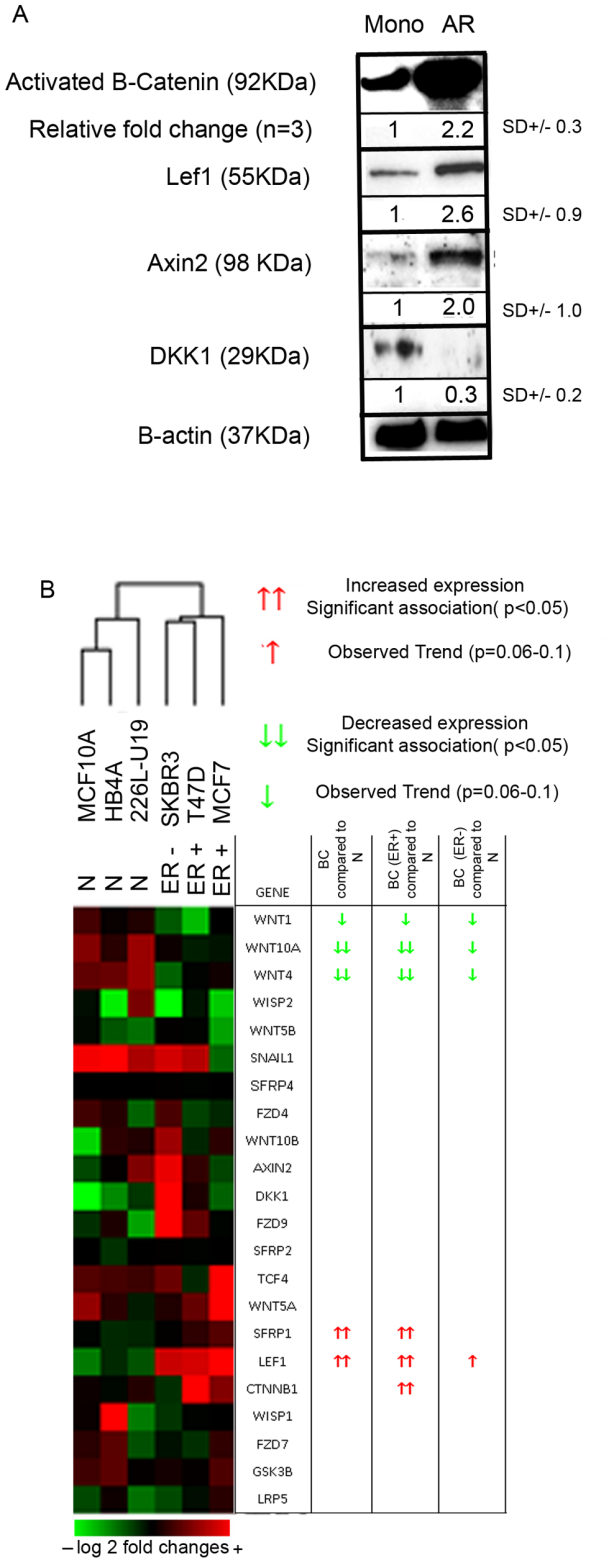
Gene expression analysis of Wnt signalling in monolayer (Mono) and anoikis resistant (AR) cells of normal breast cell lines (N), ER-ve and ER+ve breast cancer cell lines. A) Protein expression of activated B-catenin (unphosphorylated), Lef1, Axin2, DKK1 and B-actin (housekeeper) in MCF7 monolayer and AR cells. B) Cluster analysis was performed using the fold change in expression between Mono and AR cells. Data is displayed in a heatmap represented by either decreased (green) or increased (red) expression compared to the mean mRNA expression. (Red) ↑↑ Indicates significant increased expression (<0.05) (red) ↑ indicates a trend towards increased expression. (Green) Indicates significant increased expression (<0.05) (green) indicated a trend towards increased expression.

Numerous reports implicate Wnt signalling in breast stem cell activity [19], [39], [40]. To investigate this further, we employed the mammosphere culture technique to enrich for cells with stem-like activity, breast cancer stem-like cells (BCSCs). Anoikis resistant (AR) cells are enriched for stem-like activity and have increased tumour forming capacity in mice [15]. Comparison of MCF7 AR cells to adherent monolayer cells showed an increase in active WNT signalling in AR cells was confirmed by increased expression of activated beta-catenin protein, both downstream targets AXIN2, LEF1, and decreased expression of DKK1 protein (Figure 3A and Figure S2).

### Wnt Signalling is Suppressed in Normal Breast Stem-like Cells

We next investigated WNT signalling gene expression using a custom Agilent microarray using AR-derived and monolayer cultured populations from 3 immortalised normal and three breast cancer cell lines. AR-derived populations contain stem-like cells, which has been validated by in vivo tumour initiation [15;Ablett et al submitted]. Gene expression analysis confirmed results in Figure 1 that there are higher levels of WNT signalling in breast cancer cell lines compared to normal breast cell lines when grown in either monolayer or AR culture (Figure S1). Fold change differences between monolayer and AR cells (enriched for BCSCs) for each cell line were calculated and displayed as a heatmap (Figure 3B). Importantly, analysis of the change in gene expression between monolayer cultured and AR cells (enriched for BCSCs) showed differences between breast cancer and normal cell lines (Figure 3B). WNT ligands *WNT1, WNT10a* and *WNT4* were expressed at higher levels in normal AR cells compared to monolayer cells, however in the breast cancer cell lines, their expression was lower in the AR cells compared to monolayer cells. *sFRP1* and *β-catenin* were highly expressed in BCSCs from ER+ve breast cancer cell lines (MCF7 and T47D). No increase in expression was observed in the ER-ve breast cancer cell line tested (SK3RB) suggesting that canonical WNT signalling in BCSCs may correlate with ER (Figure 3B). Most importantly, we found higher levels of the WNT signalling gene *LEF1* in BCSCs compared to monolayer cells, irrespective of ER expression, while normal breast stem cell populations showed lower levels compared to normal monolayer cells (Figure 3B). The data demonstrates specific upregulation of WNT pathway signalling in a population enriched for BCSCs.

### Modulation of Wnt Pathway Signalling Affects Stem Cell-like Activity

**Figure 4:**
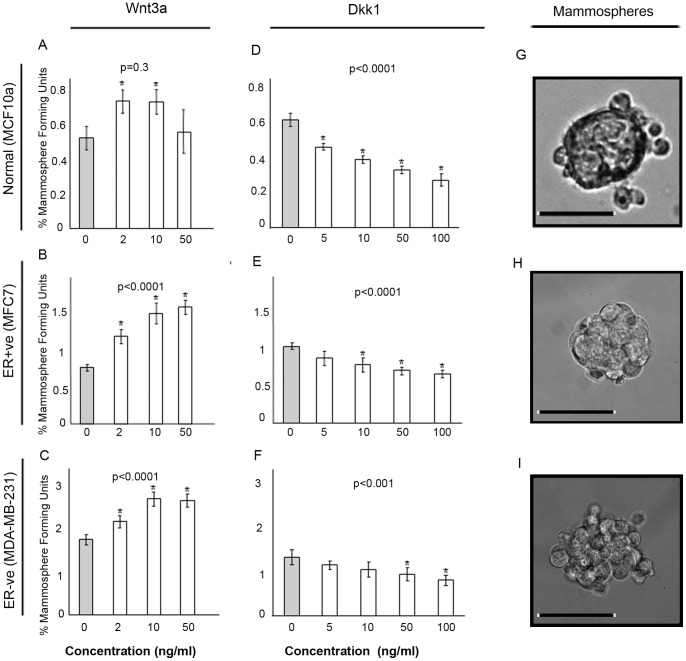
Modulation of Wnt signalling in normal and breast cancer cell lines. Single cells were plated in non-adherent conditions and treated with increasing concentrations of either Wnt3a (0–50 ng/ml) or DKK1 (0–100 ng/ml) and cultured for 7 days and number of mammospheres counted. Wnt3a treatments are displayed in the left panel and DKK1 treatments in the right panel. Light grey bars represent untreated control A) MCF10a cells (Wnt3a) B) MCF7 cells (Wnt3a) C) MDA-MB-231 cells (Wnt3a) D) MCF10a cells (DKK1) E) MCF7 cells (DKK1) F) MDA-MB-231 cells (DKK1). Data is expressed as % mammosphere formation units. P values were generated by ANOVA. Asterisks mark individual comparisons which reached statistical significance * <0.01 ** <0.001 generated by a T-test. G) Image of a MCF10a mammosphere H) Image of an MCF7 mammosphere I) Image of an MDA-MB-231 mammosphere. Scale bar represents 50 µM.

**Figure 5:**
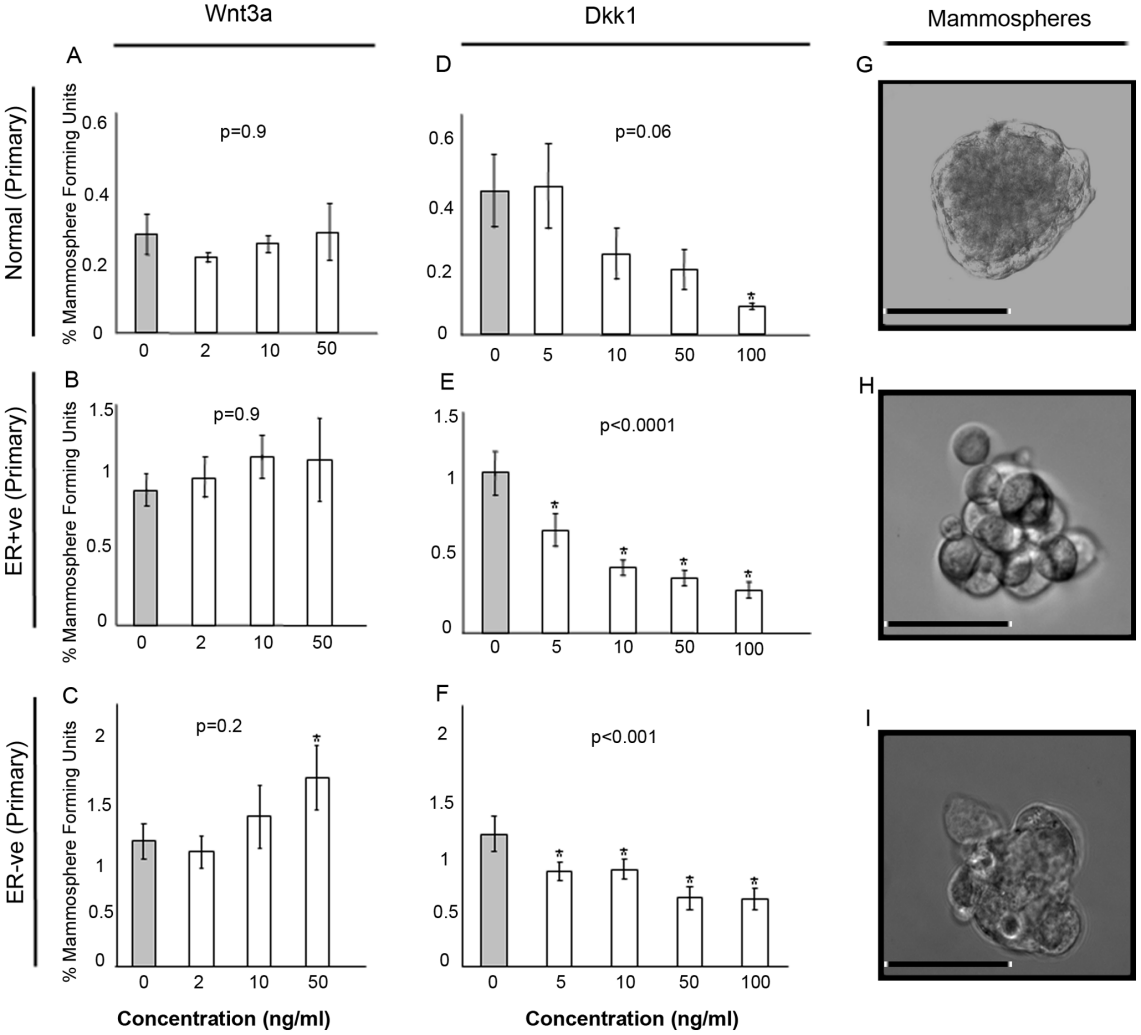
Modulation of Wnt signalling in normal and primary breast cancer samples (Normal n = 3; ER+ve n = 3; ER-ve n = 3). Single cells were plated in non-adherent conditions and treated with increasing concentrations of either Wnt3a (0–50 ng/ml) or DKK1 (0–100 ng/ml) and cultured for 7 days and number of mammospheres counted. Wnt3a treatments are displayed in the left panel and DKK1 treatments in the right panel. Light grey bars represent untreated control A) primary normal breast cells (Wnt3a) B) ER+ve primary breast cancer cells (Wnt3a) C) ER-ve primary breast cancer cells (Wnt3a) D) primary normal breast cells (DKK1) E) ER+ve primary breast cancer cells (DKK1) F) ER−ve primary breast cancer cells (DKK1). Data is expressed as % mammosphere formation units. P values were generated by ANOVA. Asterisks mark individual comparisons which reached statistical significance * >0.01 ** >0.001 generated by a T-test. G) Image of a normal primary mammosphere H) Image of an ER positive primary tumour mammosphere I) Image of an ER negative primary tumour mammosphere. Scale bar represents 50 µM.

To test the potential of targeting the WNT pathway we investigated the effects of WNT pathway modulation in cell lines. Cells were cultured as mammospheres (MS) with increasing concentrations of either DKK1 to inhibit or WNT3A to activate the WNT pathway. Recombinant WNT3A treatment significantly increased the number of MS in both normal (MCF10A) and breast cancer (MCF7 and MDA-MB-231) cell lines (Figure 4A–C). DKK1 treatment significantly decreased the number of MS in all cell lines tested with the most significant decrease observed using the highest concentration (100 ng/ml) of DKK1 (Figure 4D–F). Using patient-derived cell samples from both ER+ve (n = 3) and ER-ve (n = 3) breast cancer and normal breast tissue (n = 3) we found that WNT3A caused a significant increase in MS only in the ER-ve sample (Figure 5C). Inhibition of WNT signalling using DDK1 showed that low dose (5 ng/ml) treatment was sufficient to decrease mammosphere formation in primary breast cancer cells whilst leaving the normal cells of the breast unaffected. Only high concentrations (100 ng/ml) of DKK1 were sufficient to inhibit MS formation in normal primary breast cells (Figure 5D–F).

## Discussion

Our results are consistent with existing data [16], [41] which suggest activation of WNT signalling in breast cancer through the increased expression of key signalling components and downstream target such as *LEF1* at both the mRNA and protein level. We observed higher levels of WNT signalling in breast cancer cell lines correlating with ER expression. Although both classical downstream targets *AXIN2* and *LEF1* were on average expressed at higher levels in breast cancer cell lines, ER-ve cell lines expressed the highest levels of *AXIN2* whereas the major downstream target activated in ER+ve cell lines was *LEF1* demonstrating the subtle differences in WNT signalling that can occur.

We also observed a lack of expression of a subset of genes including *WNT5A* mRNA in ER+ve breast cancer cell lines. Recent studies suggest that *WNT5A* plays a critical role in malignant progression although loss of *WNT5A* protein has been linked with poor prognosis in breast cancer and correlated with the loss of ER expression [42]. This is contradictory to our observations of higher *WNT5A* mRNA expression in ER-ve cell lines but reported data suggests an inverse relationship between mRNA levels and expression of *WNT5A* protein [43], [44], [45].

Other genes that were inversely correlated with ER expression included *LBH*, *WISP1*, and *TCF4*. *LBH* is a newly identified target of the *WNT/β-CATENIN* signalling pathway expressed at abnormally high levels in poorly differentiated, basal-subtype, ER-ve [46]. *WISP1* is overexpressed in breast cancer and was associated with advanced clinical features [47]. *TCF4* has been reported to interact with ER signalling [48].

We next examined Wnt pathway gene expression in 1107 primary breast cancer tumours and found differences across subtypes of breast cancer. Many were associated with both basal and *ERBB2* sub-types such as *WNT5B* and others specifically in basal cancers such as *LRP5/6* and *sFRP1*. Lower levels of *sFRP1* or *LRP6* predict an early recurrence in ER+ve breast cancer whilst higher levels of *WNT5B* expression predict early recurrence. *LRP5* was the only gene that was predictive in ER-ve breast cancer where low levels predict early recurrence. The downstream target *LEF1*, which was upregulated in ER+ve cell lines, was also increased in Luminal sub-type patient samples and lower levels predicted recurrence in ER+ve patients. These analyses demonstrate the importance of Wnt signalling in breast cancer, and highlight a subgroup of ER+ve patients with a basal-type phenotype based upon their Wnt expression and decreased recurrence free survival.

Our data has shown that the WNT pathway activation is significantly higher in populations enriched for BCSCs, while populations enriched for normal stem-like cells have lower levels of WNT signalling. Furthermore, our data suggests that normal breast cancer stem cells express different WNT ligands (*WNT4* and *WNT10*) than BCSCs when compared to their monolayer populations. Little is known about the role of *WNT10A* in breast cancer but Wnt4 is essential for normal progesterone induced mammary gland development [49]. A subset of genes such as *sFRP1* and *β-CATENIN*, despite being lower in ER+ compared to ER-ve cell lines, was higher in BCSCs of ER+ve cells. This suggests that BCSCs from ER+ve cell lines share a similar expression profile to cells from an ER−ve cell line supporting the growing hypothesis of a cell hierarchy in which stem cells in ER+ve tumours are ER-ve [50]. Finally we established that the WNT pathway regulates MS formation. WNT3A treatment caused a significant increase in MS in the MCF7 and MDA-MB-231 cancer cell lines while MCF10a immortalised normal breast cells were less sensitive.

To demonstrate biologically the clinical relevance of WNT modulation, we used patient-derived breast samples from both normal and cancerous tissue and compared them to cell lines. Patient-derived samples responded less sensitively to WNT modulation and only ER−ve patient-derived breast cancer cells responded to WNT activation. Treatment with the WNT inhibitor DKK1 successfully decreased MS in both ER+ve and ER-ve patient-derived samples with most significant reduction in ER+ve cells. The data suggest that moderate DKK1 concentrations were sufficient to inhibit MS and therefore stem cell-like activity in both ER+ve and ER−ve tumours whilst not affecting the activity within normal breast tissue. This agrees with the recent in vivo studies of Gurney et al (2012), who show that a therapeutic antibody to the *FZD* receptor can prevent cancer stem cell activity without toxicity in normal stem cells [51].

### Conclusions

In conclusion, we indentified a number of novel WNT signalling components in BCSC signalling. *LEF1* has shown the most consistent activation in breast cancer cell lines and increased expression in BCSCs in both ER−ve and ER+ve patient breast cancer samples. Finally, we show that WNT pathway inhibition preferentially reduces stem-like cell activity in patient-derived metastatic breast cancer compared to normal cells. Collectively, these data suggest potential of the WNT-targeted therapeutics in breast cancer.

## Supporting Information

Figure S1 Gene expression analysis of Wnt signalling in monolayer (MO) and anoikis resistant (AR) cells of normal breast cell lines (N), ER positive (ER+) and ER negative (ER−) breast cancer cell lines. B) Cluster analysis was performed using gene expression data of cells in MO and AR. Data is displayed in a heatmap represented by either decreased (green) or increased (red) expression compared to the mean mRNA expression. (Red) ↑↑ Indicates significant increased expression (<0.05), (red) ↑ indicates a trend towards increased expression. (Green) Indicates significant increased expression (<0.05), (green) indicated a trend towards increased expression.
(TIF)

Figure S2 Full membranes used to detect Wnt signalling protein expression. Protein was quantified and gel loading volumes determined to achieve equal loading of 50 ug. Multiple gels were loaded concurrently with protein and probed for activated B-catenin (unphosphorylated), Lef1, Axin2, DKK1 and B-actin (housekeeper) in MCF7 monolayer (Day1 mono) and AR cells (Day 1 MS). B-actin was probed using Gel 1. * marks the band that represents the protein of interest.
(TIF)
