# Supplementary figures and images for: Wnt Pathway Activity in Breast Cancer Sub-Types and Stem-Like Cells
Source: PLoS One. 2013 Jul 4;8(7):e67811. doi: 10.1371/journal.pone.0067811 (PMC3701602; doi:10.1371/journal.pone.0067811)

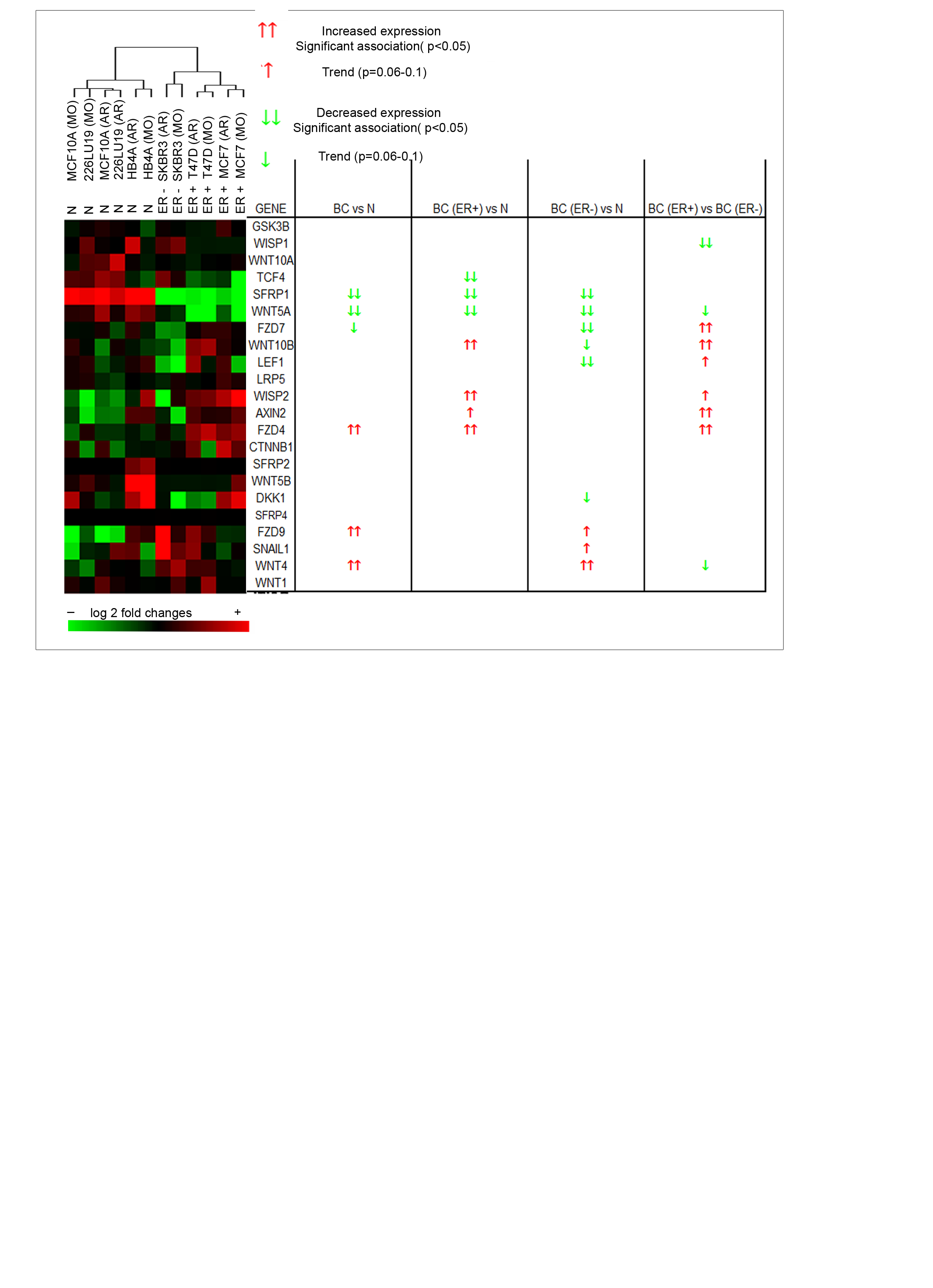

Supplement: Figure S1 — Gene expression analysis of Wnt signalling in monolayer (MO) and anoikis resistant (AR) cells of normal breast cell lines (N), ER positive (ER+) and ER negative (ER−) breast cancer cell lines. B) Cluster analysis was performed using gene expression data of cells in MO and AR. Data is displayed in a heatmap represented by either decreased (green) or increased (red) expression compared to the mean mRNA expression. (Red) ↑↑ Indicates significant increased expression (<0.05), (red) ↑ indicates a trend towards increased expression. (Green) Indicates significant increased expression (<0.05), (green) indicated a trend towards increased expression. (TIF) [file pone.0067811.s001.tif]

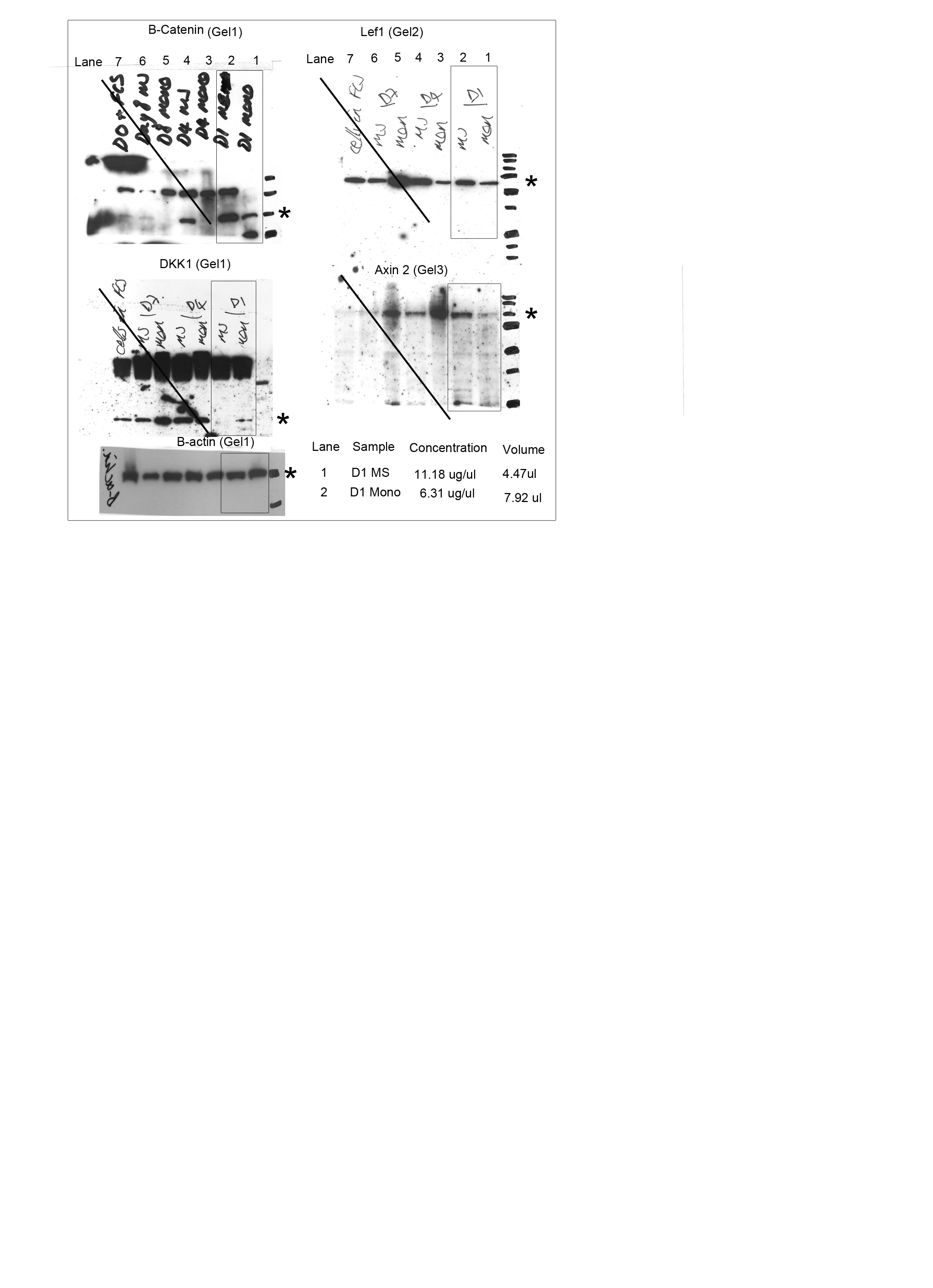

Supplement: Figure S2 — Full membranes used to detect Wnt signalling protein expression. Protein was quantified and gel loading volumes determined to achieve equal loading of 50 ug. Multiple gels were loaded concurrently with protein and probed for activated B-catenin (unphosphorylated), Lef1, Axin2, DKK1 and B-actin (housekeeper) in MCF7 monolayer (Day1 mono) and AR cells (Day 1 MS). B-actin was probed using Gel 1. * marks the band that represents the protein of interest. (TIF) [file pone.0067811.s002.tif]
